# Identification of Aerotaxis Receptor Proteins Involved in Host Plant Infection by *Pseudomonas syringae* pv. *tabaci* 6605

**DOI:** 10.1264/jsme2.ME21076

**Published:** 2022-03-10

**Authors:** Stephany Angelia Tumewu, Yuta Watanabe, Hidenori Matsui, Mikihiro Yamamoto, Yoshiteru Noutoshi, Kazuhiro Toyoda, Yuki Ichinose

**Affiliations:** 1 Graduate School of Environmental and Life Science, Okayama University, Tsushima-naka 1–1–1, Kita-ku, Okayama 700–8530, Japan; 2 The United Graduate School of Agricultural Science, Gifu University, 1–1 Yanagido, Gifu, Gifu 501–1193, Japan; 3 Faculty of Agriculture, Okayama University, Tsushima-naka 1–1–1, Kita-ku, Okayama 700–8530, Japan

**Keywords:** aerotaxis, aeroreceptor, MCP, *Pseudomonas syringae*, virulence

## Abstract

*Pseudomonas syringae* pv. *tabaci* 6605 (*Pta*6605) is a foliar plant pathogen that causes wildfire disease on tobacco plants. It requires chemotaxis to enter plants and establish infection. While chemotactic signals appear to be the main mechanism by which *Pta*6605 performs directional movement, the involvement of aerotaxis or energy taxis by this foliar pathogen is currently unknown. Based on domain structures and similarity with more than 50 previously identified putative methyl-accepting chemotaxis proteins (MCPs), the genome of *Pta*6605 encodes three potential aerotaxis transducers. We identified AerA as the main aerotaxis transducer and found that it possesses a taxis-to-serine-and-repellent (Tsr)-like domain structure that supports a periplasmic 4HB-type ligand-binding domain (LBD). The secondary aerotaxis transducer, AerB, possesses a cytosolic PAS-type LBD, similar to the Aer of *Escherichia coli* and *Pseudomonas aeruginosa*. Aerotaxis ability by single and double mutant strains of *aerA* and *aerB* was weaker than that by wild-type *Pta*6605. On the other hand, another cytosolic PAS-type LBD containing MCP did not make a major contribution to *Pta*6605 aerotaxis in our assay system. Furthermore, mutations in aerotaxis transducer genes did not affect surface motility or chemotactic attraction to yeast extract. Single and double mutant strains of *aerA* and *aerB* showed less colonization in the early stage of host plant infection and lower biofilm production than wild-type *Pta*6605. These results demonstrate the presence of aerotaxis transducers and their contribution to host plant infection by *Pta*6605.

*Pseudomonas syringae* is a foliar Gram-negative pathogenic bacterium that comprises more than 50 pathovars and, thus, causes diseases in various plant species (Mansfield *et al.*, 2012). One pathovar that causes wildfire disease in tobacco plants, *P. syringae* pv. *tabaci* 6605 (*Pta*6605), is exceptionally motile and destructive to its host plants (Ichinose *et al.*, 2013). Many factors involved in *Pta*6605 virulence have been identified, including flagellar motility, effector proteins, phytotoxins, extracellular polysaccharides, and a multidrug resistance efflux pump (Ichinose *et al.*, 2013). However, as a foliar pathogen, *Pta*6605 needs to navigate the leaf surface and enter apoplastic spaces through natural openings, such as open stomata or wounds. Flagella-mediated motility and chemotaxis play major roles in this process.

Motile bacteria perform chemotaxis as one of their sophisticated abilities to adapt to a hostile and ever-changing environment. Chemotaxis is the directed movement of cells toward a favorable life-sustaining condition (positive chemotaxis) and away from harmful substances (negative chemotaxis) (Sourjik and Wingreen, 2012). Chemotaxis requires the binding of chemical signals to the ligand-binding domain (LBD) of a chemoreceptor, a methyl-accepting chemotaxis protein (MCP), and this triggers downstream signaling that is relayed to a two-component system comprising the histidine kinase, CheA, and the response regulator, CheY, which regulates flagellar rotation (Bi and Lai, 2015). Bacterial chemotaxis has been extensively examined in model organisms such as *Escherichia coli* and *P. aeruginosa* (Parkinson *et al.*, 2015; Sampedro *et al.*, 2015). We recently investigated the role of genes encoding the core chemotaxis proteins (CheA and CheY) for *Pta*6605 virulence, and found that chemotaxis-defective mutants were less motile and unable to establish an infection (Tumewu *et al.*, 2021b).

Aerotaxis, often called energy taxis, is performed by bacterial cells in response to the ever-changing environment (Schweinitzer and Josenhans, 2010). Although aerotaxis signaling induces a behavioral response to oxygen, its complex signaling mechanisms have not yet been elucidated. When bacteria inhabit a microenvironment, the level of oxygen will continue to decrease during bacterial multiplication. The use of oxygen triggers electron transport, which, in turn, regulates the proton motive force. To guide bacterial cells toward oxygen, aerotaxis transducer proteins sense increasing levels of electron transport or the proton motive force (Rebbapragada *et al.*, 1997; Edwards *et al.*, 2006). Similar to chemotaxis, an aerotaxis signaling pathway was reported to require a signaling relay mediated by CheA, CheY, the coupling protein CheW, and a dedicated MCP-like transducer protein (Rowsell *et al.*, 1995). Downstream signaling upon ligand sensing is highly similar to chemotaxis, as described above.

MCPs function as specific sensors for various physicochemical signals. An MCP typically has an LBD, transmembrane domains (TMDs), a histidine kinase, adenyl cyclase, methyl-accepting chemotaxis protein and phosphatase (HAMP) domain, and a signaling domain (SD) (Ud-‍Din and Roujeinikova, 2017). The ligand-sensing specificity of an MCP is generally influenced by the type of LBD. Chemoreceptor proteins may be grouped into seven types based on the localization of the LBD and the presence and number of TMDs (Ud-Din and Roujeinikova, 2017). The aerotaxis transducer in *E. coli*, Aer, which senses the redox status of the electron transport system, has been intensively investigated. Aer contains a cytoplasmic PAS-type LBD and is embedded in the cell membrane by TMDs (Edwards *et al.*, 2006; Fig. 1D). This topological organization is common in other aerotaxis transducers, such as Aer of *Pseudomonas putida* (Nichols and Harwood, 2000) and *P. aeruginosa* (Hong *et al.*, 2004), Aer-1 of *Vibrio cholerae* (Boin and Häse, 2007), Aer-1 and Aer-2 of *Ralstonia solanacearum* (Yao and Allen, 2007), and Aer1-1 and Aer1-2 of *Pseudomonas chlororaphis* (Arrebola and Cazorla, 2020). On the other hand, atypical Aer transducers were also found in AerC of *Azospirillum brasilense* (Xie *et al.*, 2010) and Aer-2 of *V. cholerae* (Boin and Häse, 2007) and *P. aeruginosa* (Hong *et al.*, 2004). Due to the absence of TMDs, these Aer transducers are soluble proteins that are expected to localize in the cytoplasm. This type of Aer consists of a HAMP domain, PAS domain, and SD. The PAS domain of the Aer transducers described above is associated with a flavine adenine dinucleotide (FAD) cofactor, which allows them to sense changes in the redox status of the electron transport system. On the other hand, the taxis to serine and repellent (Tsr) of *E. coli* does not possess a PAS-type LBD; it supports a periplasmic 4-helix-bundle (4HB)-type LBD. Tsr presumably senses the proton motive force directly or indirectly (Rebbapragada *et al.*, 1997).

Chemotaxis plays an important part in infection and disease development caused by animal and plant pathogens (Matilla and Krell, 2018). In plant-infecting bacteria, chemotaxis towards plant-derived compounds is considered to facilitate entry into plants through natural openings or wounds (Matilla and Krell, 2018). Aerotaxis itself has been associated with effective and efficient attachment and colonization by the biocontrol bacteria *A. brasilense* (Greer-Phillips *et al.*, 2004) and *P. chlororaphis* (Arrebola and Cazorla, 2020) and the soil-borne pathogen *R. solanacearum* (Yao and Allen, 2007). We assume that *Pta*6605, as a foliar plant pathogen, also performs aerotaxis to reach a potential niche that supports its survival on the harsh foliar plane. However, there is currently no information on aerotaxis by foliar plant pathogens. Therefore, we herein identified two functional aerotaxis transducers in *Pta*6605, AerA and AerB. AerA showed similarity to the Tsr of *E. coli*, while AerB resembled the typical PAS-domain-containing transmembrane Aer proteins. Aerotaxis is also required for early colonization in host plants and plays a role in the formation of biofilms by this foliar plant pathogen.

## Materials and Methods

### Bacterial strains and growth conditions

*P. syringae* pv. *tabaci* 6605 strains were maintained in King’s B medium supplemented with nalidixic acid (Nal) (Shimizu *et al.*, 2003). *E. coli* strains used in DNA recombination and mutant construction were maintained in Luria-Bertani (LB) medium supplemented with appropriate antibiotics. The bacterial strains and plasmids used in the present study are listed in Table 1.

### Generation of predicted aerotaxis mutants and complemented strains

To evaluate the function of *aer* gene products in *Pta*6605, several series of mutant strains were constructed. The genetic regions of A3SK_RS0109810 (hereinafter referred to as RS0109810), A3SK_RS0105130 (RS0105130), and A3SK_RS0118845 (RS0118845), including the upstream and downstream regions, were amplified using a series of primer pairs and isolated using a TA cloning system (pGEM T-Easy; Promega) to obtain pG-RS0109810, pG-RS0105130, and pG-RS0118845, respectively. All primers used in the present study are listed in Table 2. Inverse PCR was performed to delete each open reading frame (ORF) using each set of primers. After transformation, the deleted constructs of the plasmids obtained, pG-ΔRS0109810, pG-ΔRS0105130, and pG-ΔRS011884, were confirmed by PCR and DNA sequencing. Mutated DNA fragments were excised and subcloned into pK18*mobsacB* (Schäfer *et al.*, 1994), and the resultant plasmids, pK18-ΔRS0109810, pK18-ΔRS0105130, and pK18-ΔRS0118845, were introduced into *E. coli* S17-1. Mutations were introduced by the conjugation of *Pta*6605 wild-type (WT) and plasmid-possessing *E. coli* S17-1 with subsequent homologous recombination, and each deletion mutant was selected on King’s B agar plates supplemented with 10% sucrose. To define each mutant, we assigned names to the encoding genes as follows: *aerA* for RS0109810, *aerB* for RS0105130, and *aerC* for RS0118845. Each deletion mutant, Δ*aerA*, Δ*aerB*, and Δ*aerC*, was confirmed by PCR.

Regarding the *aerA* gene, we generated not only a deletion mutant, but also a deoxyadenosine monophosphate (A) insertion mutant. Using pG-RS0109810 as a template and two complementary mutated oligonucleotides, as shown in Table 2, the site-directed mutation of RS0109810 was introduced by PCR and pG-RS0109810m was generated. Conjugation and homologous recombination were performed using *Pta*6605 WT and *E. coli* S17-1 possessing pK18-RS0109810m. After 10% sucrose selection, we performed colony PCR to amplify RS0109810 genomic DNA and digested the PCR product using *Hin*dIII. The *Hin*dIII site was newly generated by the insertion of an “A”. This insertion caused a frameshift mutation, and the 87^th^–88^th^ amino acids were changed to alanine and a stop codon (Fig. S1).

To generate the double mutant of *aerA*/Δ*aerB*, a mutation in Δ*aerB* was introduced into an *aerA*-defective mutant strain. To complement this mutation, the full-length *aerA* gene was subcloned into the transposon vector pBSL118 at the *Eco*RI site (Alexeyev and Shokolenko, 1995). The expression vector pDSK519 (Keen *et al.*, 1988) was used to subclone the *aerB* gene at the *Eco*RI site. Constructs were transformed into the *E. coli* S17-1 λpir strain (Simon *et al.*, 1983) for conjugation to the respective mutant strains. The resulting strains were screened by PCR and were resistant to Nal and kanamycin (Nal^r^Km^r^).

### Aerotaxis assay

Aerotaxis was assayed based on the air trap scheme of Arrebola and Cazorla (2020) with several modifications. Before the experiment, modified Pasteur pipettes and 3‍ ‍mL of LB medium containing 0.1% agar in sterile glass test tubes were prepared. Bacterial strains were precultured in KB liquid medium supplemented with Nal for approximately 20 h. Bacterial cells were washed twice by centrifugation and resuspended in 10‍ ‍mM of HEPES buffer (pH 7.4) at an OD_600_ of 1.0. The bacterial suspension (150‍ ‍μL) was injected into the LB broth outside the Pasteur pipette. To create an air trap inside the Pasteur pipette, 1‍ ‍mL of paraffin oil was carefully overlaid on the LB broth outside the Pasteur pipette. A schematic representation of the experimental set-up is shown in Fig. 2A. Test tubes were incubated at 27°C for 24 h without any agitation. Photographs were taken after the incubation, 150‍ ‍μL of the medium inside the Pasteur pipette was then collected, and bacterial populations were counted.

### Surface motility assays

To investigate the surface motility of *aer* mutant strains, motility assays were performed on semisolid agar medium as previously described (Tumewu *et al.*, 2020). Bacterial strains were cultured overnight in LB broth supplemented with 10‍ ‍mM MgCl_2_ and resuspended in 10‍ ‍mM of MgSO_4_ at an OD_600_ of 0.1. In the swimming assay, 3‍ ‍μL of the bacterial suspension was spotted onto the center of 0.25% agar minimal medium (MM; 50‍ ‍mM potassium phosphate buffer, 7.6‍ ‍mM (NH_4_)_2_SO_4_, 1.7‍ ‍mM MgCl_2_ and 1.7‍ ‍mM NaCl, pH 5.7) supplemented with 10‍ ‍mM each of mannitol and fructose (MMMF) and then incubated at 23°C for 72 h. The same amount of the bacterial suspension was spotted onto SWM plates (0.45% agar, 0.5% peptone, and 0.3% yeast extract; Difco) and incubated at 27°C for 48 h for a swarming assay. Photographs were taken at the end of the incubation.

### Chemotaxis assay

A quantitative chemotaxis assay was performed as described in our previous study (Tumewu *et al.*, 2020). To prepare bacterial cells, each strain was cultured in LB medium supplemented with 10‍ ‍mM MgCl_2_ overnight. After washing with MM, bacterial cells were resuspended in MMMF broth and incubated for another 5‍ ‍h to starve cells. After the incubation, cells were washed and resuspended in 10‍ ‍mM of HEPES buffer (pH 7.4) at an OD_600_ of 0.05. To prepare attractant-containing capillaries, one end of a glass capillary (5‍ ‍μL; Drummond Scientific) was sealed with a flame and dipped in attractant solution. Prepared capillaries were submerged in 200‍ ‍μL of the bacterial suspension in a 96-well microtiter plate. After a 30-min incubation at 27°C, capillaries were washed, and the content was pushed out using a dedicated plunger. Bacterial colony-forming units (CFUs) were then counted after serial dilutions and plating on KB with Nal plates.

### Biofilm formation assay

To quantify the ability of cells to attach to a polypropylene surface, a biofilm assay was performed. Each strain was cultured overnight in LB broth with 10‍ ‍mM MgCl_2_. Bacterial cells were washed and resuspended in MMMF broth at an OD_600_ of 0.1. The bacterial suspension (200‍ ‍μL) was dispensed in a polypropylene microtiter plate (Corning) and incubated at 27°C for 48 h without shaking. Cells that adhered to the polypropylene microtiter plate were stained with crystal violet, and the pigment in stained cells was extracted by 95% ethanol. Absorbance was measured at 595‍ ‍nm using an iMark™ Microplate Absorbance Reader (Bio-Rad Laboratories) (Tumewu *et al.*, 2021a).

### Host plant and virulence assay

The virulence of WT and mutant strains was analyzed using a flood inoculation method as described in our previous study (Tumewu *et al.*, 2020). Briefly, gas-sterilized tobacco seeds (*Nicotiana tabacum* L. cv. Xanthi NC) were sown on Murashige-Skoog (MS) plates supplemented with 1% sucrose and vitamin solution (thiamin hydrochloride 3‍ ‍mg L^–1^, nicotinic acid 5‍ ‍mg L^–1^, and pyridoxine hydrochloride 0.5‍ ‍mg L^–1^). After a 2-week incubation, seedlings were transplanted into 0.1% sucrose MS plates and grown for a further 3 d. The inoculum was prepared in 10‍ ‍mM MgSO_4_ at an OD_600_ of 0.004 (8×10^6^ CFU mL^–1^) supplemented with 0.025% (v/v) Silwet L-77 (OSI Specialties) to promote cell attachment. Tobacco seedlings were flooded by the inoculum for approximately 10 s. The inoculum was decanted, and the plate was left to dry inside a clean bench for 15‍ ‍min. After 6 h and 3‍ ‍d of the incubation at 22°C (with a long photoperiod), 2 leaf disks (Ø 4‍ ‍mm) were collected and ground in 1‍ ‍mL of sterilized water. After serial dilutions, the suspension was spotted on KB agar in Nal plates. Recovered bacterial CFUs were assessed after a 2-d incubation at 27°C. The fresh weight of the seedlings (mg) was also measured at 6‍ ‍d post-inoculation (dpi). The aerial part of each seedling was cut and gently blotted with a paper towel to remove excess moisture and then immediately weighed. Photos of seedlings were taken at 3 and 6 dpi.

### Data ana­lysis

The significance of differences in aerotaxis ability, chemotaxis ability, biofilm formation, and bacterial populations between mutant strains and WT was examined using an ana­lysis of variance (ANOVA), followed by Dunnett’s test at the 95% confidence level. Analyses of all data were performed using GraphPad Prism ver. 9 (GraphPad Software). *P*<0.05 was considered to be significant.

## Results

### Predicted aerotaxis transducer genes in *Pta*6605

**Fig. 1.**
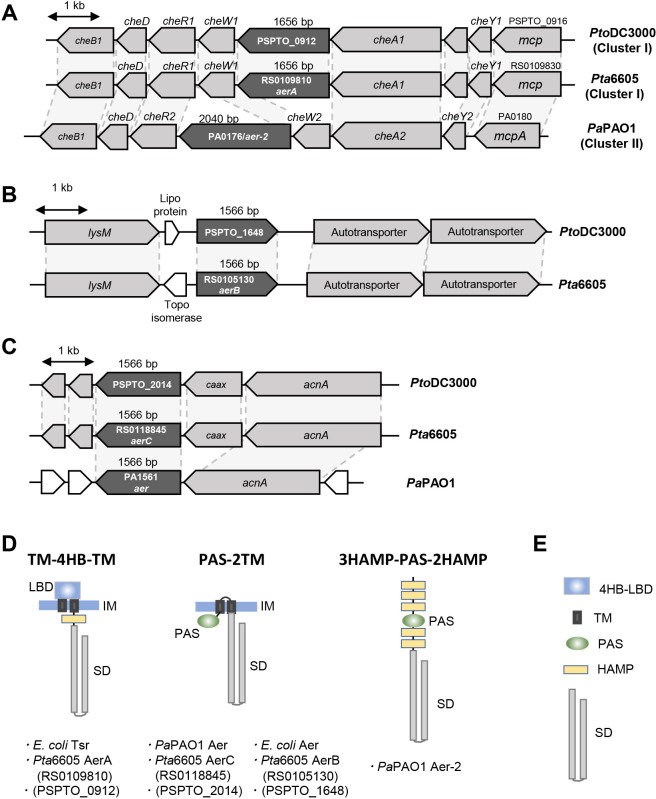
Predicted *aer* transducer genes of *Pta*6605 and their homologs in the closely related plant pathogen *Pto*DC3000 and animal pathogen *Pa*PAO1. Potential *aer* transducer genes were depicted by dark gray pentagons and each ortholog is connected with a shadow background. Schematic organization of *che* gene clusters including RS0109810 (*aerA*) (A), RS0105130 (*aerB*) (B), RS0118845 (*aerC*) (C), and the surrounding genes in *Pta*6605, and their orthologs in *Pto*DC3000 and *Pa*PAO1. (D) Schematic model of the domain organization of predicted Aer transducer proteins in *Pta*6605 and reference proteins in *E. coli* and *Pa*PAO1. Each domain is explained in E. (E) Blue rectangles indicate the 4HB-type LBD; small black rectangles indicate transmembrane domains (TMDs), green ovals indicate the PAS-type LBD; cream rectangles indicate HAMP domains, and two consecutive gray rectangles indicate signaling domains (SDs).

*Pta*6605 has more than 50 genes encoding putative MCPs in its genome. Since *P. aeruginosa* and *P. syringae* strains conserve high DNA sequence synteny, we searched for the corresponding orthologs of the *aer* and *aer-2* genes of *P. aeruginosa* PAO1 (*Pa*PAO1) (Hong *et al.*, 2004) in *Pta*6605. The *aer* gene (PA1561) in *Pa*PAO1 was located downstream of *acnA* (gene encoding aconitate hydratase 1), and the *mcp* genes PSPTO_2014 in *P. syringae* pv. *tomato* DC3000 (*Pto*DC3000) and RS0118845 in *Pta*6605 were located in similar loci; however, a single gene encoding the CAAX amino terminal protease family protein was located between the *mcp* and *acnA* genes in *P. syringae* (Fig. 1C). Amino acid identities among Aer in *Pa*PAO1, MCPs encoded by PSPTO_2014, and RS0118845 were more than 77%, and MCPs encoded by PSPTO_2014 and RS0118845 showed 96.4% amino acid identity. Aer also showed 50% amino acid similarity to MCPs encoded by PSPTO_1648 and RS0105130, and MCPs encoded by PSPTO_1648 and RS0105130 showed 96.0% amino acid identity to each other. Furthermore, PSPTO_1648 and RS0105130 were identified at the same gene locus (Fig. 1B). The MCPs described above had a similar overall structure. Although Aer of *Pa*PAO1 showed homology to many MCPs in *Pto*DC3000 and *Pta*6605, homology was restricted in the SD. The deduced structures of the above proteins belongs to the “PAS-2TM-SD” type of the MCP family, which, in turn, belongs to topology type II by the classification of Ud-Din and Roujeinikova (2017). Originally named PAS for three different eukaryotic proteins, Per-ARNT-Sim (period, hydrocarbon receptor nuclear translocator, single-minded) is cytosolic and diverse in many organisms, and includes a transducer molecule that senses light, oxygen, and redox signals (Schweinitzer and Josenhans, 2010; Ortega *et al.*, 2017). Aer in *E. coli* was also shown to conserve the PAS-2TM-SD structure (Rebbapragada *et al.*, 1997); therefore, the structure of Aer of *Pa*PAO1 resembles that of Aer of *E. coli* (Bibikov *et al.*, 2000), although the latter has a HAMP domain before the SD (Fig. 1D). There were no other genes encoding “PAS-2TM-SD” type MCP in *Pto*DC3000 or *Pta*6605.

*Pa*PAO1 has been shown to possess another aerotaxis transducer gene, *aer-2* (Hong *et al.*, 2004). The *aer-2* gene (PA0176) was located on the chemotaxis gene cluster as one ORF of a large operon (Fig. 1A). This gene cluster in *Pa*PAO1 was well conserved in *Pto*DC3000 and *Pta*6605, and the deduced amino acid sequences in each ortholog were very similar, except for one; however, gene names (number) and cluster numbers were defined in a different manner. In the corresponding locus of the *aer-2* gene of *Pa*PAO1, we found the *mcp* genes, PSPTO_0912 in *Pto*DC3000 and RS0109810 in *Pta*6605 (Fig. 1A). However, the predicted structures of Aer-2 of *Pa*PAO1 and MCPs encoded by PSPTO_0912 and RS0109810 were very different, and neither *Pto*DC3000 nor *Pta*6605 had similar genes to the *aer-2* gene in any genomic area. Aer-2 of *Pa*PAO1 was classified as a topology type IVa MCP (Ud-Din and Roujeinikova, 2017), and the structure was very unusual. Aer-2 of *Pa*PAO1 is a cytosolic protein composed of three HAMP domains followed by a PAS domain, two HAMP domains, and the SD (3HAMP-PAS-2HAMP-SD) (Watts *et al.*, 2011). PSPTO_0912 and RS0109810 both encoded topology type Ia, and the LBD type was 4HB (Fig. 1D). The topology type Ia MCP harbored a periplasmic LBD, two TM domains, and a cytosolic SD. Since the topology types of Aer-2 of *Pa*PAO1 and MCPs encoded by *Pto*DC3000 and *Pta*6605 differed, amino acid identity was restricted in only the SD to approximately 40%. The structures of MCPs encoded by PSPTO_0912 and RS0109810 resembled the Tsr of *E. coli*. Tsr is an energy taxis sensor that responds to serine, carbon sources, pH, temperature, and oxygen (Schweinitzer and Josenhans, 2010). Therefore, in addition to RS0105130 (*aerB*) and RS0118845 (*aerC*), RS0109810 (*aerA*) may be a gene that encodes a potential aerotaxis transducer in *Pta*6605.

### Aerotaxis of WT and *aer* mutant strains in *Pta*6605

We initially generated deletion mutants of the *aerA*, *aerB*, and *aerC* genes. In the aerotaxis assay, although a high density of the WT strain was observed inside the Pasteur pipette in contact with air, the Δ*aerA* mutant was not present, indicating that the positive aerotaxis activity of the WT strain was lost in the Δ*aerA* mutant (Fig. S2A). Furthermore, the Δ*aerA* mutant abolished both surface swarming and swimming motilities and did not cause disease. However, when the intact *aerA* gene was introduced into the Δ*aerA* mutant, the complemented strain did not restore the WT phenotype in any experiment (Fig. S2A). We also conducted a chemotaxis assay to a known attractant, yeast extract, and found that neither the Δ*aerA* mutant nor Δ*aerA*-C exhibited chemotaxis activity (Fig. S2B). Therefore, the cause of the altered phenotype in the Δ*aerA* mutant was not the loss of AerA function; this phenotype may be due to a polar effect of the deletion of the *aerA* gene. Therefore, the *aerA* mutant was regenerated by the introduction of a stop codon in the *aerA* ORF.

**Fig. 2.**
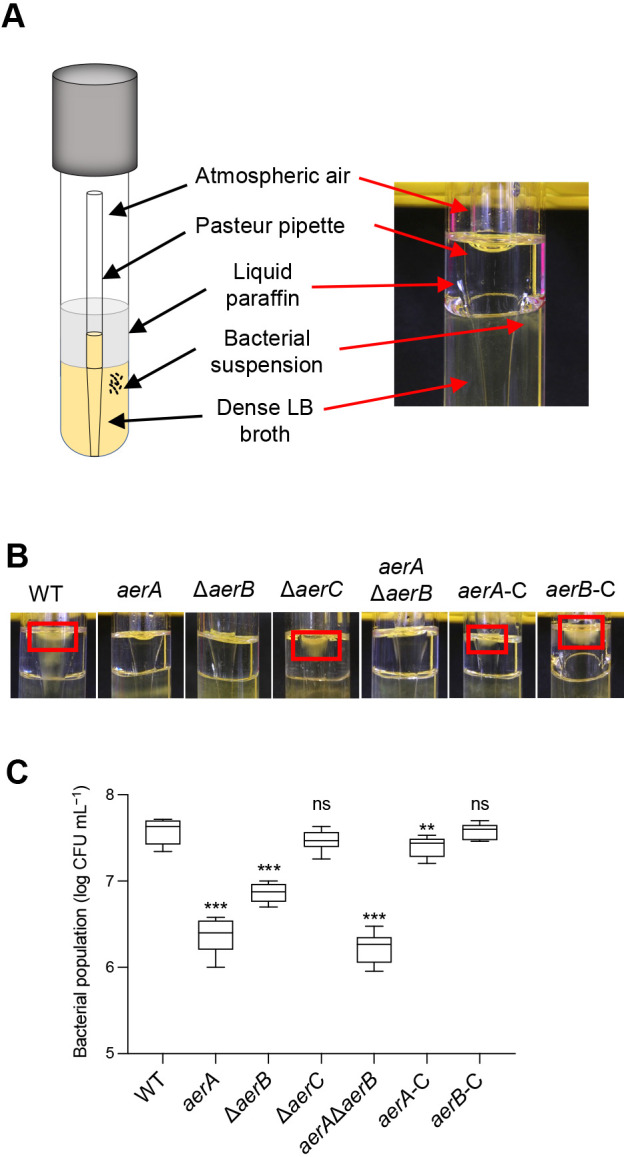
Aerotaxis assay of WT, *aer* mutant, and complemented strains of *Pta*6605. (A) Experimental set-up for the aerotaxis assay. Using a test tube and modified Pasteur pipette containing dense LB broth, a bacterial suspension was inoculated outside the pipette in addition to liquid paraffin to create an air-trap environment. (B) Qualitative observation of aerotaxis assay results after a 24-h static incubation at 27°C. Red squares refer to dense bacteria in LB medium that are in contact with air inside the Pasteur pipette. (C) Quantitative ana­lysis of bacterial populations moved to LB medium in contact with air inside the Pasteur pipette. The bacterial population was expressed as log CFU mL^–1^. Asterisks indicate significant differences between WT and mutant strains at ***P<0.001 or **P<0.01 by Dunnett’s multiple comparison test. Error bars represent standard errors from two independent experiments conducted in triplicate. ns: not significant.

Aerotaxis by the *aerA*, Δ*aerB*, and Δ*aerC* mutant strains was investigated. As shown in Fig. 2A, dense bacteria were observed in the upper medium in contact with air for the WT and Δ*aerC* mutant strains, but not for the *aerA*, Δ*aerB*, or *aerA*/Δ*aerB* mutant strains. Furthermore, the accumulation of bacterial cells was noted for the complemented strains *aerA*-C and Δ*aerB*-C (Fig. 2A). To quantitatively analyze the level of aerotaxis, the bacterial population was measured (Fig. 2B). The level of impairment in aerotaxis was more severe for the *aerA* mutant and *aerA*/Δ*aerB* mutant strains, while that for the Δ*aerB* mutant was mild. The bacterial population of the Δ*aerC* mutant was similar to that of the WT strain, and the bacterial populations of *aerA*-C and Δ*aerB*-C had almost recovered to the WT level.

### Aerotaxis and chemotaxis signal transduction pathway

**Fig. 3.**
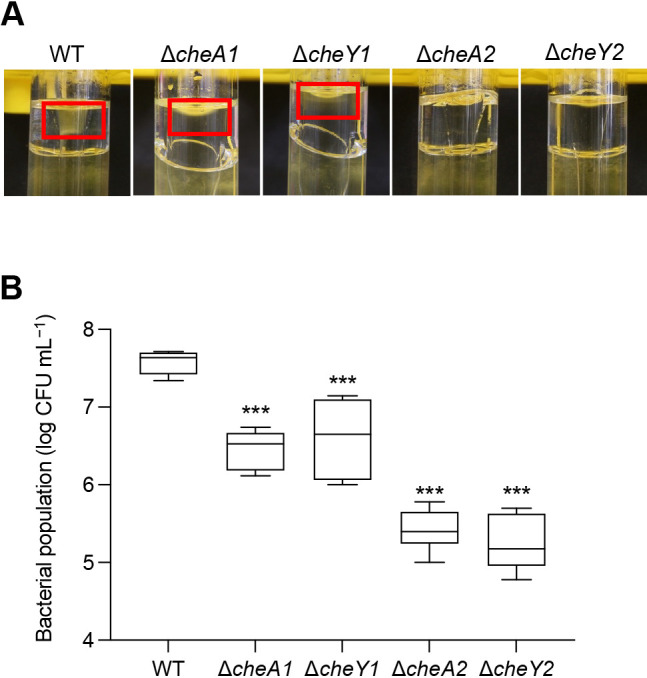
Aerotaxis assay of the WT strain and *che* mutant strains of *Pta*6605. (A) Qualitative observation of aerotaxis assay results after a 24-h static incubation at 27°C. Red squares refer to dense bacteria in the LB medium that are in contact with air inside the Pasteur pipette. (B) Quantitative ana­lysis of bacterial populations that moved to LB medium in contact with air inside the Pasteur pipette. The bacterial population was expressed as log CFU mL^–1^. Asterisks indicate significant differences between the WT and mutant strains at ***P<0.001 by Dunnett’s multiple comparison test. Error bars represent standard errors from two independent experiments conducted in triplicate.

It currently remains unclear whether *Pta*6605 needs a general chemotaxis signal transduction pathway to perform aerotaxis. Therefore, we examined the requirement for a two-component system (CheA and CheY) using four mutant strains (Δ*cheA1*, Δ*cheA2*, Δ*cheY1*, and Δ*cheY2*; Tumewu *et al.*, 2021b) from the two major chemotaxis clusters in the aerotaxis assay. Aerotaxis to atmospheric air was examined using the air trap scheme described in the Methods section. Δ*cheA2* and Δ*cheY2* mutants, lacking a functional cluster II two-component system, did not move into the Pasteur pipette, in which the surface of the LB medium was in contact with air (Fig. 3A). Aerotaxis by the cluster I mutants, Δ*cheA1* and Δ*cheY1*, was reduced, but not to the same extent as that by cluster II mutants. This result was also quantitatively supported. Significantly fewer bacterial CFU were recovered from medium inside the Pasteur pipette for the Δ*cheA2* and Δ*cheY2* strains than for the WT strain (Fig. 3B). In contrast, more bacterial CFU were recovered for the Δ*cheA1* and Δ*cheY1* mutants than for the Δ*cheA2* and Δ*cheY2* mutants, but less than WT. These results suggest that aerotaxis by *Pta*6605 requires the signal to be processed by the general chemotaxis pathway.

### Surface motility and chemotaxis of *aerA*, *aerB*, and *aerC* mutants

**Fig. 4.**
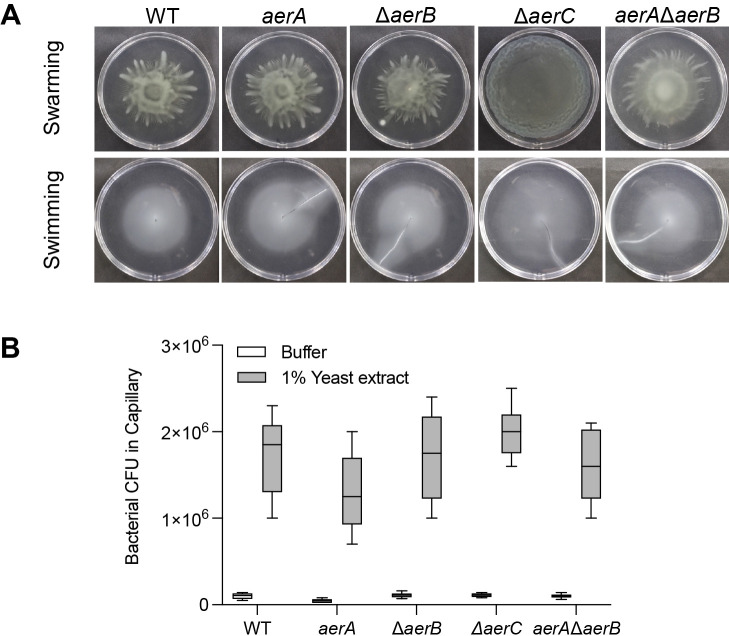
Surface motility and chemotaxis of the WT strain and *aer* mutant strains of *Pta*6605. (A) Surface swarming motility was assessed on 0.45% agar SWM plates and swimming motility in 0.25% agar MMMF plates. Photographs were taken at 48 hpi for swarming plates and at 72 hpi for swimming plates. Photographs shown are representative of three biological repeats each with three technical replicates. (B) Quantitative chemotaxis assay towards 1% yeast extract solution. Bacterial cells that move towards the attractant in capillaries were measured on KB plates supplemented with Nal. No significant differences were observed between the WT and mutant strains by Dunnett’s multiple comparison test. Error bars indicate standard errors from two independent experiments conducted in triplicate.

To rule out the possibility that general motility by the mutants was impaired, we examined their surface motility and chemotactic attraction to yeast extract. Swarming and swimming motilities were investigated on 0.45% agar SWM plates and 0.25% agar MMMF plates, respectively. As shown in Fig. 4A, WT, the *aerA* mutant, Δ*aerB* mutant, and double mutant showed similar swarming patterns on SWM plates, while the Δ*aerC* mutant produced a rather smooth pattern without the formation of tendrils. On swimming MMMF plates, all strains showed similar motility, except for the Δ*aerC* mutant, which had a larger swimming diameter (Fig. 4A). A chemotaxis assay to 1% yeast extract was conducted using a quantitative capillary method (Tumewu *et al.*, 2021b). All mutant strains were attracted to yeast extract, similar to the WT strain (Fig. 4B), suggesting that mutations in aerotaxis transducer genes did not significantly affect general motility or the core chemotaxis system.

### Virulence assay

**Fig. 5.**
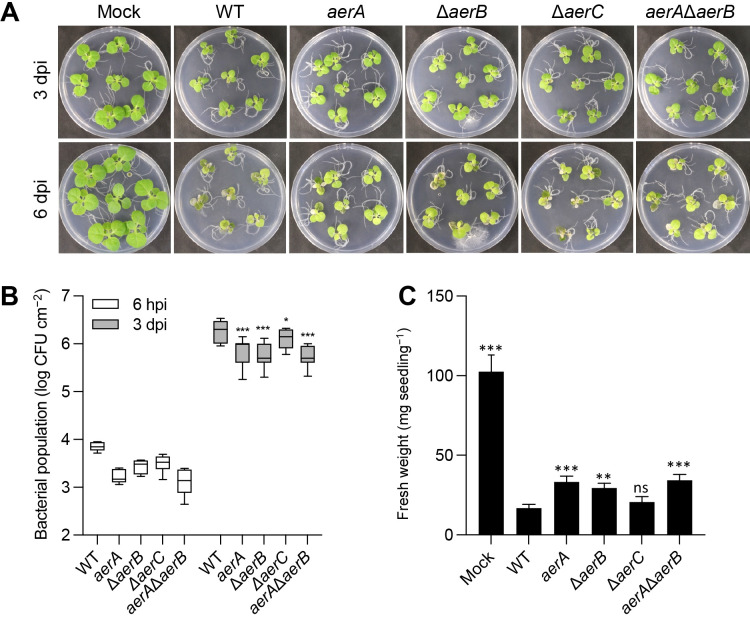
Flood inoculation assay of WT strain and *aer* mutant strains of *Pta*6605 on host tobacco seedlings. (A) Symptom development of wildfire disease on host tobacco seedlings at 3 dpi and 6 dpi. The inoculum was 8×10^6^ CFU mL^–1^ of each bacterial strain, and the incubation was performed at 22°C with a long photoperiod. (B) Bacterial populations recovered from the inoculated seedlings at 6 hpi (white boxes) and 3 dpi (gray boxes). Error bars represent standard errors from two independent experiments with 3 seedlings for 6 hpi and 7 seedlings for 3 dpi. Asterisks indicate significant differences from WT at each time point by Dunnett’s multiple comparison test (*P<0.05; ***P<0.001). (C) Fresh weight of seedlings at 6 dpi. Error bars represent the standard error of the mean from 2 biological repeats each with 7 individual seedlings. Asterisks indicate significant differences from WT by Dunnett’s multiple comparison test (ns: not significant; **P<0.01; ***P<0.001).

The *Pta*6605 chemotaxis system plays a significant role in virulence (Tumewu *et al.*, 2021b). Furthermore, mutations in the *mcp* genes for γ-aminobutyric acid and amino acids had a prominent impact on *Pta*6605 fitness in host plants (Tumewu *et al.*, 2020; 2021a). To elucidate the role of aerotaxis on virulence by *Pta*6605, a flood inoculation assay was performed. As shown in Fig. 5A, WT and mutant strains still caused severe disease symptoms, although the seedlings inoculated with the *aerA* mutant, Δ*aerB* mutant, and double mutant strains appeared to be slightly bigger than those inoculated with WT. Therefore, we measured bacterial populations that successfully entered the leaves as well as the fresh weight of seedlings. Fig. 5B shows that at 6‍ ‍h post-inoculation (hpi), fewer bacterial populations were recovered from seedlings inoculated with mutant strains than with WT; however, these differences were not significant. Differences in the *aerA* mutant, Δ*aerB* mutant, and *aerA*/Δ*aerB* mutant were significant at 3 dpi. Except for the Δ*aerC* mutant, the fresh weights of seedlings inoculated with the mutant strains were approximately 2-fold those inoculated with the WT strain (Fig. 5C). This result suggests that aerotaxis plays a minor, but important role in the early stage of *Pta*6605 invasion into a host plant.

### Biofilm formation

**Fig. 6.**
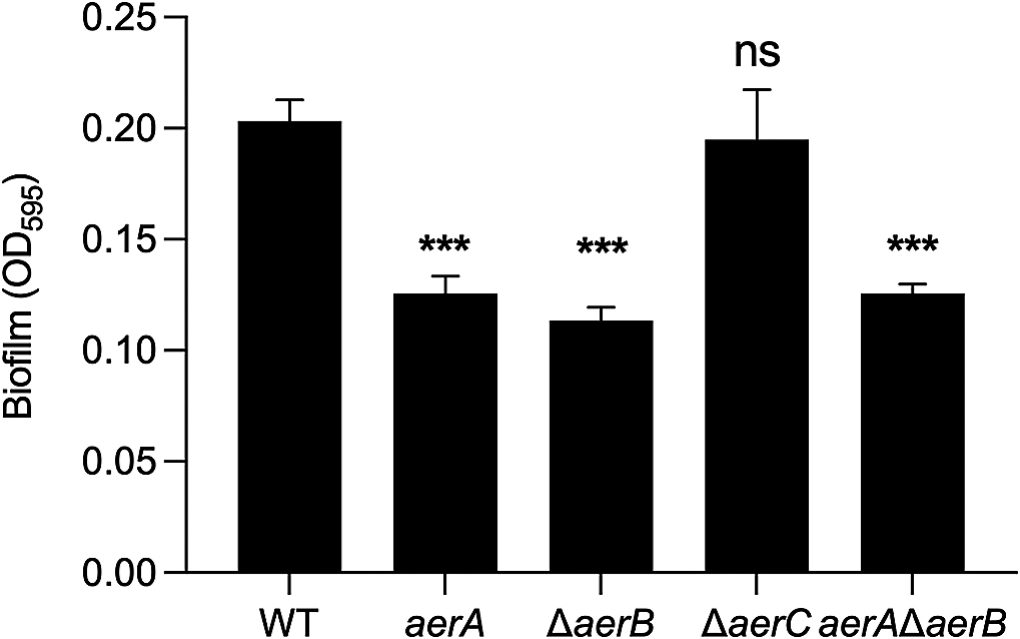
Biofilm formation by WT and *aer* mutant strains of *Pta*6605. Biofilms were stained by crystal violet after a 48-h static incubation at 27°C. Quantification was performed by measuring the OD_595_ of a stained biofilm extracted with 95% ethanol. Error bars indicate standard errors from two independent experiments with five replicates. Asterisks indicate significant differences from the WT strain analyzed by Dunnett’s multiple comparisons test (***P<0.001). ns: not significant.

*Pta*6605 forms a biofilm when transitioning from a planktonic to sessile lifestyle (Ichinose *et al.*, 2013). As previously reported, aerotaxis contributed to pellicle formation when bacterial cells formed cell aggregates or a biofilm on medium-air contact surfaces (Hölscher *et al.*, 2015). The present results revealed marked differences in biofilm densities between the *aerA*, Δ*aerB*, and *aerA*/Δ*aerB* mutants and the WT strain after a 48-h static incubation at 27°C (Fig. 6), indicating the role of aerotaxis in biofilm regulation in *Pta*6605.

## Discussion

### Characterization of aerotaxis transducer proteins in *Pta*6605

Among the approximately 50 putative chemoreceptor proteins in *Pta*6605, AerB and AerC are membrane-anchored MCPs with intracellular PAS-type LBD similar to the typical aerotaxis transducers Aer of *E. coli*, *P. putida*, and *P. aeruginosa* (Hong *et al.*, 2004; Taylor, 2007). AerB and AerC showed 50.4 and 78.5% amino acid sequence identities, respectively, to Aer of *P. aeruginosa*, while AerB and AerC showed 50.2% amino acid sequence identity to each other. Although AerB exhibited aerotaxis activity, AerC did not, at least under the conditions used in the present study, and did not appear to be involved in *Pta*6605 aerotaxis. Nevertheless, since it is a PAS-type MCP, AerC may be another energy taxis chemoreceptor because AerC showed high identity (78.5%) to Aer of *Pa*PAO1. Other PAS-type LBD-containing cytoplasmic MCPs have been reported, such as AerC of *A. brasilense* and Aer-2 of *Pa*PAO1 (Hong *et al.*, 2004; Xie *et al.*, 2010). AerC of *A. brasilense* is a 2×PAS-SD-type MCP that functions as a redox sensor to support nitrogen fixation (Xie *et al.*, 2010). On the other hand, as described above, Aer-2 of *Pa*PAO1 is composed of 3HAMP-PAS-2HAMP-SD. Although *Pta*6605 does not have an ortholog of Aer-2 of *Pa*PAO1, *Pta*6605 has four genes encoding cytoplasmic 2×PAS-SD type MCP (A3SK_RS0101665, A3SK_RS0107145, A3SK_RS0111330, and A3SK_RS0121510) and a gene encoding 4×PAS-SD type MCP (A3SK_RS0112230). Further studies are needed to clarify whether these intracellular PAS-SD-type MCPs respond to oxygen.

AerA has a periplasmic 4HB-type LBD (Fig. 1D). An MCP with 4HB-LBD is the most common MCP in *Pta*6605, and there are at least 16 genes encoding this type of MCP. McpS in *P. putida* and CtpH in *P. aeruginosa* are 4HB-LBD-possessing MCPs that are responsible for sensing TCA cycle intermediates (Pineda-Molina *et al.*, 2012) and inorganic phosphate (Wu *et al.*, 2000), respectively. Therefore, MCPs with periplasmic 4HB-type LBD appear to sense particular chemical compounds. However, Tsr with the 4HB-type LBD (Fig. 1D) is a secondary aerotaxis transducer in *E. coli* and directly or indirectly senses changes in proton motive force (Kim *et al.*, 1999). Tsr not only senses oxygen (Rebbapragada *et al.*, 1997), it responds to serine (Rebbapragada *et al.*, 1997), carbon sources (Greer-Phillips *et al.*, 2003), pH (Umemura *et al.*, 2002), and temperature (Lee *et al.*, 1988). Aer and Tsr in *E. coli* are both capable of sensing oxidizable carbon sources (Greer-Phillips *et al.*, 2003). Therefore, it is plausible that AerA in *Pta*6605 functions as an aerotaxis transducer. The MCP of *Pta*6605, which has the highest amino acid identity for Tsr, is AerA. Although the identity of the amino acid sequence is not very high (32.8%), the homologous region is not only an SD, it is also the entire protein structure. Another reason why we investigated AerA is due to the location of the gene in the *Pta*6605 genome. As described in the Results section, the *aerA* gene was located in *che* cluster I; the homologs of *che* cluster II of *P. aeruginosa* and the locus of the *aerA* gene of *Pta*6605 and *aer-2* gene of *Pa*PAO1 were the same (Fig. 1A).

### Involvement of the chemotaxis signal transduction pathway in aerotaxis

Chemotaxis gene clusters (*che*) generally comprise genes encoding proteins that are crucial for the signal transduction pathway of chemotaxis (Wuichet and Zhulin, 2010). Although Aer-mediated aerotaxis is independent of CheR and CheB methylation, the signal perceived by an Aer transducer is still able to induce CheA autophosphorylation and presumably affect downstream processes (Bibikov *et al.*, 2004). Therefore, the presence of core chemotaxis proteins, such as CheA or CheY, may also be crucial for aerotaxis. In *E. coli*, CheA, CheW, and CheY are required for aerotaxis (Rowsell *et al.*, 1995), and the presence of *cheYZABW* genes in *che* cluster I of *Pa*PAO1 (orthologs of *che* cluster II in *Pta*6605) was confirmed to be essential for aerotaxis (Hong *et al.*, 2004). Aerotaxis assays using four *cheA* and *cheY* mutant strains revealed that the group III chemotaxis gene cluster (cluster I in *Pa*PAO1 and cluster II in *Pta*6605) was dominant in both chemotaxis and aerotaxis, and that the group II chemotaxis gene cluster (cluster II in *Pa*PAO1 and cluster I in *Pta*6605) played a minor role in both chemotaxis and aerotaxis (Tumewu *et al.*, 2021b and Fig. 3).

The deletion of the *aerA* gene resulted in the loss of aerotaxis ability, chemotaxis ability, swarming and swimming motilities, and virulence (Fig. S2). Since the *aerA* mutant retained swarming and swimming motilities, the phenotype of the Δ*aerA* mutant is the result of a polar effect. The *aerA* gene and *cheA1* gene belong to the same *che1* cluster in *Pta*6605 (Fig. 1A). We also found that the loss of virulence in the Δ*cheA1* mutant of the same *che1* cluster was not recovered by complementation of the *cheA1* gene, indicating that a DNA sequence containing the *cheA1* gene and *aerA* gene is required for the transcription and/or RNA stability of the *che1* cluster in *Pta*6605 (Tumewu *et al.*, 2021b).

The reduced aerotactic abilities of *aerA* and Δ*aerB* mutants were not due to impaired motility or chemotaxis because these mutant strains retained the same level of surface motility and chemotaxis as the WT strain (Fig. 4).

### Importance of aerotaxis in the virulence of the foliar pathogen *Pta*6605

Yao and Allen (2007) previously demonstrated the significant role of aerotaxis by Aer1 and Aer2 in the interaction between *R. solanacearum* and its host tomato plant. The *aer2* mutant or *aer1*/*aer2* mutant slightly delayed wilt disease on host tomato plants, indicating that *R. solanacearum* needs aerotaxis for normal interactions with host plants. However, *R. solanacearum* is a soilborne pathogen, and the role of aerotaxis or energy taxis for foliar plant pathogens, such as *Pta*6605, has yet to be investigated. In the present study, we identified two functional aerotaxis transducers in *Pta*6605. Our air trap scheme experiment revealed that *Pta*6605 WT moved toward and gathered at the medium-air contact point inside the Pasteur pipette, as observed in the photograph and bacterial population in Fig. 2. Populations of the *aerA* mutant, Δ*aerB* mutant, and double mutant strains were significantly smaller than that of the WT strain, indicating that AerA and AerB both contribute to aerotaxis. The population of the *aerA* mutant was slightly smaller than that of the Δ*aerB* mutant, indicating that AerA is the predominant transducer of aerotaxis.

In plant-infecting bacteria, chemotaxis towards plant-derived compounds is considered to facilitate entry into the plant through natural openings or wounds. Flagella-mediated motility is important for *Pta*6605 virulence because chemotaxis-defective mutants showed a significantly weaker ability to cause disease symptoms than WT (Tumewu *et al.*, 2021b). Some chemoreceptors are associated with virulence, such as the GABA chemoreceptor McpG (Tumewu *et al.*, 2020) and amino acid chemoreceptors PscB and PscC2 (Tumewu *et al.*, 2021a). We hypothesized that aerotaxis transducers also play this role in the interaction between *Pta*6605 and its host plants. The *Pta*6605 *aerA* mutant, Δ*aerB* mutant, and double mutants caused slightly different disease symptoms (Fig. 5A). The bacterial populations of the mutant strains at 3 dpi were significantly smaller than that of WT, which may have been because fewer bacterial cells had entered the seedlings at the earlier time (Fig. 5B). In comparisons with the non-motile and avirulent Δ*cheA2* mutant and Δ*cheY2* mutant (Tumewu *et al.*, 2021b), the non-aerotactic *aerA* mutant and Δ*aerB* mutant were still capable of causing severe disease symptoms, as observed at 6 dpi (Fig. 5A), and the marked decrease in the fresh weight of tobacco seedlings after the inoculation with the WT strain was slightly improved after the inoculation with the *aerA* mutant and Δ*aerB* mutant (Fig. 5C). The presence of many other MCPs in *Pta*6605 governing other types of taxis may facilitate the entry of pathogens into tobacco seedlings. Similar cases of biocontrol ability in *P. chlororaphis* and virulence in *R. solanacearum* were observed (Yao and Allen, 2007; Arrebola and Cazorla, 2020).

Plant pathogenic *Pseudomonas* produces a biofilm structure to ensure survival in a harsh environment (Heredia-Ponce *et al.*, 2021). *Pta*6605 forms a biofilm and attaches to the host plant surface, providing a safe space to further the infection process by producing toxins and effectors. Biofilm formation by *Pta*6605 is regulated through various complex pathways, including flagella and pili-mediated motilities (Ichinose *et al.*, 2013). The non-aerotactic *aerA* mutant, Δ*aerB* mutant, and double mutant strains showed less attachment, as indicated by the lower biofilm biomass on an abiotic surface (Fig. 6). Combined with the lower bacterial population found inside the seedlings, we inferred that the ability to form a biofilm may help *Pta*6605 attach to the host surface and effectively cause disease. The regulation of biofilm formation appears to differ among bacterial species. An aerotaxis mutant of the soilborne pathogen *R. solanacearum* overproduced biofilms (Yao and Allen, 2007), whereas biofilm formation by the Δ*aer1-1* and Δ*aer1-2* mutants of the rhizobacterium *P. chlororaphis* was reduced (Arrebola and Cazorla, 2020).

In the present study, we demonstrated the requirement of *aerA* and *aerB*, genes for aerotaxis transducers, for the interaction of the foliar pathogen *Pta*6605 with its host tobacco plants. We presumed that aerotaxis helps *Pta*6605 to navigate the foliar surface and reach oxygen-rich stomata openings, at which gas exchange occurs. Following entry into the plant, bacterial cells attach to the cell surface, initiating the next infection process. However, the absence of aerotaxis affects bacterial entry and attachment because bacterial cells presumably do not recognize the space as a ‘suitable’ place to settle. *Pta*6605 has approximately 50 other MCPs with various LBD types that sense many chemical signals coming from plants, which helps bacterial cells to adapt and cause disease. Further investigations are necessary.

## Citation

Tumewu, S. A., Watanabe, Y., Matsui, H., Yamamoto, M., Noutoshi, Y., Toyoda, K., and Ichinose, Y. (2022) Identification of Aerotaxis Receptor Proteins Involved in Host Plant Infection by *Pseudomonas syringae* pv. *tabaci* 6605. *Microbes Environ ***37**: ME21076.

https://doi.org/10.1264/jsme2.ME21076

## Supplementary Material

Supplementary Material

## Figures and Tables

**Table 1.** Bacterial strains and plasmids used in the present study

Bacterial strain, plasmid	Relevant characteristics	Reference or source
*Escherichia coli*		
DH5α	*F^–^λ^–^ ϕ80dLacZ *Δ*M15 *Δ(*lacZYA-argF*)*U169 recA1 endA1 hsdR17*(*rK*^–^* mK^+^*)* supE44 thi-1 gyrA relA1*	Nippon Gene
S17-1	*thi pro hsdR hsdR hsdM^+^ recA*(*chr::RP4-2-Tc::Mu-Km::Tn7*)	Schäfer *et al.* 1994
S17-1 λpir	λpir lysogeny of S17-1	Simon *et al.* 1983
*P. syringae* pv. *tabaci*		
Isolate 6605	Wild-type isolated from tobacco, fully virulent, Nal^r^	Shimizu *et al.* 2003
Δ*aerA*	Isolate 6605 ΔRS0109810, Nal^r^	This study
Δ*aerA*-C	pB-*aerA* introduced *aerA*, Nal^r^ Km^r^	This study
*aerA*	Isolate 6605 insertional mutant in RS0109810, Nal^r^	This study
*aerA*-C	pB-*aerA* introduced *aerA*, Nal^r^ Km^r^	This study
Δ*aerB*	Isolate 6605 ΔRS0105130, Nal^r^	This study
Δ*aerB*-C	pD-*aerB* possessing Δ*aerB*, Nal^r^ Km^r^	This study
Δ*aerC*	Isolate 6605 ΔRS0118845, Nal^r^	This study
*aerA*Δ*aerB*	Isolate 6605 double mutation in RS0109810 and RS0105130, Nal^r^	This study
Plasmid		
pGEM-TEasy	Cloning vector, Amp^r^	Promega
pG-RS0109810	RS0109810 fragment-containing pGEM-T Easy, Amp^r^	This study
pG-ΔRS0109810	ΔRS0109810 fragment-containing pGEM-T Easy, Amp^r^	This study
pG-RS0109810m	Mutated RS0109810 fragment-containing pGEM-T Easy, Amp^r^	This study
pG-RS0105130	RS0105130 fragment-containing pGEM-T Easy, Amp^r^	This study
pG-ΔRS0105130	ΔRS0105130 fragment-containing pGEM-T Easy, Amp^r^	This study
pG-RS0118845	RS0118845 fragment-containing pGEM-T Easy, Amp^r^	This study
pG-ΔRS0118845	ΔRS0118845 fragment-containing pGEM-T Easy, Amp^r^	This study
pK18*mob*Sac*B*	Small mobilizable vector, Km^r^, sucrose sensitive (s*acB*)	Schäfer *et al.* 1994
pK18-ΔRS0109810	RS0109810 deleted DNA-containing pK18*mobsacB*, Km^r^	This study
pK18-RS0109810m	Mutated RS0109810 fragment-containing pK18*mobsacB*, Km^r^	This study
pK18-ΔRS0105130	RS0105130 deleted DNA-containing pK18*mobsacB*, Km^r^	This study
pK18-ΔRS0118845	RS0118845 deleted DNA-containing pK18*mobsacB*, Km^r^	This study
pDSK519	Broad host range cloning vector, Km^r^	Keen *et al.* 1988
pBSL118	Mini-Tn5-derived plasmid vector for insertion mutagenesis, Amp^r^, Km^r^	Alexeyev *et al.* 1995
pB-*aerA*	pBSL118 possessing expressible *aerA*, Km^r^	This study
pD-*aerB*	pDSK519 possessing expressible *aerB*, Km^r^	This study

Nal^r^, nalidixic acid-resistant; Amp^r^, ampicillin-resistant; Km^r^, kanamycin-resistant.

**Table 2.** Primers used in the present study

Primer Name	Sequence (5′--3′)	Description
RS0109810_a	GCTGACGCTGGCGATCATC	Amplification of RS0109810 ORF for insertional mutagenesis
RS0109810_b	GGTGACTTTGGCGTTCTCGG	
RS0109810_1	AGTACGTGATGTCAGTCAGG	Amplification of RS0109810 and the surrounding region
RS0109810_2	AACCGACCACTTCCCAAGG	
RS0109810_3	CTAgctagcTCCTTGGGAATTGCGAATCCG	Deletion of RS0109810 ORF
RS0109810_4	CTAgctagcCGGCTTCGATAGAGACTCCA	
RS0109810_5	CCAGGAAAAGGCGCAGATGG**A**AGCTTGAAGCAGCGGAC	Insertional mutagenesis of RS0109810
RS0109810_6	GTCCGCTGCTTCAAGCT**T**CCATCTGCGCCTTTTCCTGG	
RS0109810_7	GGGTTCGATCCTTGAACAGTGCAGC	Amplification of RS0109810 and the promoter region for complementation
RS0109810_8	TCAGGGCAGGATCAGCTTGGAAACC	
RS0105130_1	TTACAGTGCGGACACGCTGG	Amplification of RS0105130 and the surrounding region
RS0105130_2	CCAAATGGAGTCTGCGTTACGG	
RS0105130_3	CGCggatccGGTTCAGTCGCTAAGCATGC	Deletion of RS0105130 ORF
RS0105130_4	CGCggatccATAGGCATGTTGACGCGCAT	
RS0118845_1	CCTCGCATTGGCCTTTCATC	Amplification of RS0118845 and the surrounding region
RS0118845_2	TGGCGCAAGCAGTGCTGC	
RS0118845_3	GAagatctGACTTGAGACTGTTTTCAACGCC	Deletion of RS0118845 ORF
RS0118845_4	GAagatctTGGGTTTCGATCCTTCGATC	

Bold letters indicate artificial nucleotides for the insertional mutagenesis of RS0109810 ORF in RS0109810_5 and RS0109810_6. Lowercase letters indicate the artificial nucleotide sequence for *Nhe*I in RS0109810_3 and RS0109810_4, *Bam*HI in RS0105130_3 and RS0105130_4, and *BgI*II in RS0118845_3 and RS0118845_4.
