# Supplementary material for: Identification of Aerotaxis Receptor Proteins Involved in Host Plant Infection by Pseudomonas syringae pv. tabaci 6605
Source: Microbes Environ. 2022 Mar 10;37(1):ME21076. doi: 10.1264/jsme2.ME21076 (PMC8958299; doi:10.1264/jsme2.ME21076)
Supplement: Supplementary file 1 — Supplementary Material [file 37_21076_s1.pdf]

```
          10        20        30        40        50        60
ATGAAATGGTTCTACGATCTGAAGATTTCCACCAAGCTGATTACCTCGTTTCTGGTGGTT
 M  K  W  F  Y  D  L  K  I  S  T  K  L  I  T  S  F  L  V  V

          70        80        90       100       110       120
CTGGCGTTGACCGCGGCCATGGGAGTCTTTGCCATCATCCAGCTCGGCCAGGTCAACCAG
 L  A  L  T  A  A  M  G  V  F  A  I  I  Q  L  G  Q  V  N  Q

         130       140       150       160       170       180
GCCGCTCAGGACATCAAGGAAAACTGGATGCCGTCCATGCGCGCGGCTTCGGGCATGCGC
 A  A  Q  D  I  K  E  N  W  M  P  S  M  R  A  A  S  G  M  R

         190       200       210       220       230       240
TTCTTCGCCGCCAACTATCGCTTGAAGGAAAACCGCCACATTGCAGCCGACACCGCCCAG
 F  F  A  A  N  Y  R  L  K  E  N  R  H  I  A  A  D  T  A  Q

         250       260       270       280       290       300
GAAAAGGCGCAGATGGAAGCTTGAAGCAGCGGACTCTCGCAAACAGTTCGAAACCCGCCT
 E  K  A  Q  M  E  A  *
```

Fig. S1. Site-directed mutagenesis to introduce stop codon and *Hin*dIII site in *aerA* gene. Partial nucleotide sequence of open reading frame from translation start codon with corresponding amino acid sequence below the nucleotide sequence is shown. Position of oligonucleotide used in PCR is underlined. An artificially inserted extra “A” and mutated amino acid (A) are shown in red. Newly generated *Hin*dIII site and stop codon are shown in italic and blue, respectively.

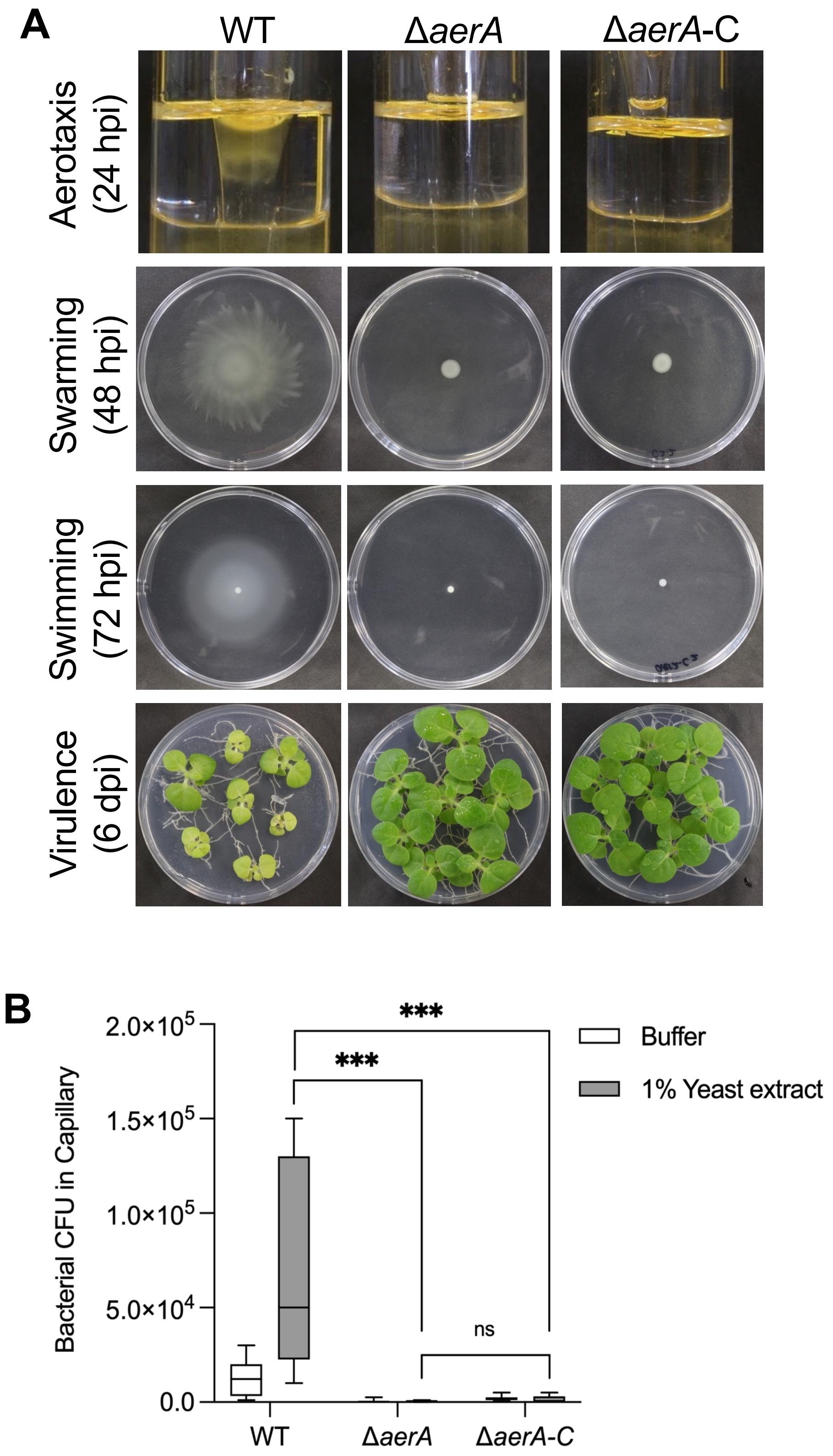

Fig. S2. Phenotypes of Δ*aerA* mutant strain compared to WT and its complemented strain (Δ*aerA*-C) of *Pta*6605. (A) Δ*aerA* mutant was impaired in aerotaxis, surface swarming and swimming motilities, and virulence by flood inoculation assay. Complemented strain of Δ*aerA* mutant showed the same phenotype as Δ*aerA* mutant. (B) The Δ*aerA* mutant and Δ*aerA*-C lost chemotaxis activity to 1% yeast extract. Asterisks indicate statistically significant differences compared to WT strain analyzed by Tukey's multiple comparisons test (****P* < 0.001). ns: not significant.
